# H19 long noncoding RNA alters trophoblast cell migration and invasion by regulating TβR3 in placentae with fetal growth restriction

**DOI:** 10.18632/oncotarget.9534

**Published:** 2016-05-21

**Authors:** Lisa Zuckerwise, Jing Li, Lingeng Lu, Yi Men, Tingting Geng, Catalin S. Buhimschi, Irina A. Buhimschi, Radek Bukowski, Seth Guller, Michael Paidas, Yingqun Huang

**Affiliations:** ^1^ Department of Obstetrics, Gynecology and Reproductive Sciences, Yale University School of Medicine, New Haven, CT, USA; ^2^ Deparment of Obstetrics and Gynecology, Nanfang Hospital, Southern Medical University, Guangzhou, Guangdong, P.R. China; ^3^ Department of Chronic Diseases Epidemiology, Yale School of Public Health, Yale University School of Medicine, New Haven, CT, USA; ^4^ Department of Head and Neck Surgery, State Key Laboratory of Oral Diseases, West China Hospital of Stomatology, Sichuan University, Chengdu, Sichuan, P.R. China; ^5^ Department of Endocrinology, School of Medicine, First Affiliated Hospital of Xi'an Jiaotong University, Xi'an, Shaanxi, P.R. China; ^6^ Department of Obstetrics and Gynecology, The Ohio State University College of Medicine, Columbus, OH, USA; ^7^ Center for Perinatal Research, The Research Institute at Nationwide Children's Hospital, Columbus, OH, USA; ^8^ Department of Pediatrics, The Ohio State University College of Medicine, Columbus, OH, USA; ^9^ Yale Women and Children's Center for Blood Disorders and Preeclampsia Advancement, Yale School of Medicine, New Haven, CT, USA

**Keywords:** fetal growth restriction, H19 long noncoding RNA, TβR3, trophoblast

## Abstract

Fetal growth restriction (FGR) is a well-recognized risk factor for perinatal mortality and morbidity, as well as neurodevelopmental impairment and adulthood onset disorders. Here we report that the H19 long noncoding RNA (lncRNA) is significantly decreased in placentae from pregnancies with FGR. Downregulation of H19 leads to reduced migration and invasion of extravillous trophoblast (EVT) cells *in vitro*. This is consistent with reduced trophoblast invasion that has been observed in FGR. Genome-scale transcriptome profiling of EVT cells reveals significantly decreased expression of the type III TGF-β receptor (TβR3) following H19 knockdown. Decreased TβR3 expression is also seen in FGR placentae. TβR3 repression decreases EVT cell migration and invasion, owing to impaired TGF-β signaling through a non-canonical TGF-β signaling pathway. Further, we identify TβR3 as a novel regulatory target of microRNA let-7. We propose that dysregulation of this newly identified H19/TβR3-mediated regulatory pathway may contribute to the molecular mechanism of FGR. Our findings are the first to show a lncRNA-based mechanism of FGR, holding promise for the development of novel predictive, diagnostic, and therapeutic modalities for FGR.

## INTRODUCTION

Fetal growth restriction (FGR), defined as estimated fetal weight below the 10th percentile, identifies a group of fetuses who are at risk for perinatal and longer term mortality and morbidity [1, 2]. Perinatal morbidity associated with FGR includes neonatal encephalopathy, cerebral palsy, sepsis, seizures, respiratory distress, prolonged hospitalization and need for intensive care unit admission. These risks increase with severity of growth restriction. In preterm infants with FGR, a large retrospective study of over one million neonates found an increased risk of respiratory distress syndrome, intraventricular hemorrhage, and necrotizing enterocolitis as compared to appropriately sized gestational-age matched neonates [3]. The increased morbidity associated with FGR extends beyond the neonatal period. FGR is associated with increased risk of cardiovascular and endocrine disorders in adulthood [4]. Additionally, children from pregnancies with FGR have demonstrated significantly lower academic achievement in school as well as lower professional achievement in adulthood [5, 6]. The molecular mechanisms underlying FGR remain in the majority of cases poorly understood.

Proper placental development is essential for fetal growth and survival. The extravillous trophoblast (EVT), a subset of placental cells, plays a critical role in this process. EVT cells migrate and invade into the uterine wall, leading to remodeling of the maternal vasculature. This remodeling yields a low-resistance, high-capacity perfusion system that allows for adequate exchange of oxygen, nutrients and key molecules at the maternal-fetal interface. The migratory and invasive properties of EVT are stringently controlled and derangement can lead to pathological conditions. Indeed, suboptimal trophoblast invasion has been associated with FGR and preeclampsia, whereas excessive trophoblast invasion has been linked to placenta accreta and choriocarcinoma (reviewed in [7, 8]).

The *H19* gene encodes a polyadenylated, long noncoding RNA (lncRNA) of 2,600-nt that predominantly resides in the cytoplasm but with a minor fraction also found in the nucleus [9, 10]. *H19* is abundantly expressed both in placentae and embryos, and is strongly down-regulated after birth in most adult tissues [9]. Mechanistically, it has been shown that H19 lncRNA (herein called H19) in the nucleus recruits repressive histone markers to differentially methylated regions of a group of imprinted network genes, thereby inhibiting their transcription and contributing to embryo growth regulation in the mouse [10]. Nuclear H19 also serves as precursor for microRNA miR-675 in a cell/tissue-specific and developmentally regulated manner [11–13]. In the mouse, through the action of miR-675, *H19* maintains adult hematopoietic stem cells [12], promotes skeletal muscle differentiation and regeneration [13], and results in the physiological inhibition of placental growth just before birth [11]. In the cytoplasm, H19 acts as a molecular sponge for microRNA let-7 thereby reducing its bioavailability and preventing it from repressing target gene expression at the posttranscriptional level [14]. Through this action, H19 plays a role in regulation of muscle cell differentiation [14], glucose metabolism [15], tumor metastasis [16], and endometrial development [17].

Altered imprinting or epigenetic regulation of the *H19*–*Igf2* locus in human has been associated with altered placental and fetal growth as well as pregnancy complications ([18, 19], and reviewed in [20]). However, the underlying molecular mechanism of H19's role in placentation remains poorly defined. Using *in situ* hybridization it was shown that *H19* is highly expressed in human placental intermediate trophoblast, cytotrophoblast (including EVT), and syncytiotrophoblast tissue [21], suggesting an important role for *H19* in trophoblast physiology.

In this report, we investigate a mechanism through which H19 participates in the pathogenesis of FGR. We find that H19 is significantly decreased in human placentae with idiopathic FGR. We provide evidence that depressed H19 reduces TGF-β signaling through a non-canonical pathway activated by TβR3, leading to impaired migration and invasion of EVT cells. We propose that dysregulation of this newly identified H19/TβR3-mediated regulatory pathway may contribute to the underlying mechanism of idiopathic FGR.

## RESULTS

### H19 promotes EVT migration and invasion by inhibiting microRNA let-7

We have previously reported that H19 promotes migration and invasion of tumor cells by decreasing the bioavailability of microRNA let-7 [16]. Bearing multiple binding sites for let-7, H19 binds to and sequesters let-7, preventing it from repressing target gene expression at the posttranscriptional level [14]. Given the abundance of H19 in the EVT [21], and its known role in regulating migration and invasion, we hypothesized that H19 might function to regulate EVT development. Thus, effects of H19 repression on EVT were evaluated using HTR-8/SVneo (called HTR herein), a cell line derived from human first trimester EVT [22]. H19 was knocked down using siRNA (siH19, [14, 16]) in the presence and absence of a let-7 inhibitor (iLet7, [14, 16]), followed by analysis of cell migration and invasion using transwell assays. iLet7s are chemically modified oligonucleotides that specifically bind to let-7 and inhibit its activity. The rationale for including iLet7 was to confirm the contribution of let-7 to the H19-mediated pathway, as H19 has other functions besides sequestering let-7 [10–13, 23]. Thus, in the presence of iLet7, we would expect that the effects of H19 knockdown on EVT would be attenuated, as iLet7 would neutralize let-7 released from H19 sequestration. The ability of iLet7 to relieve inhibition of other let-7 targets has been previously documented [15–17].

**Figure 1:**
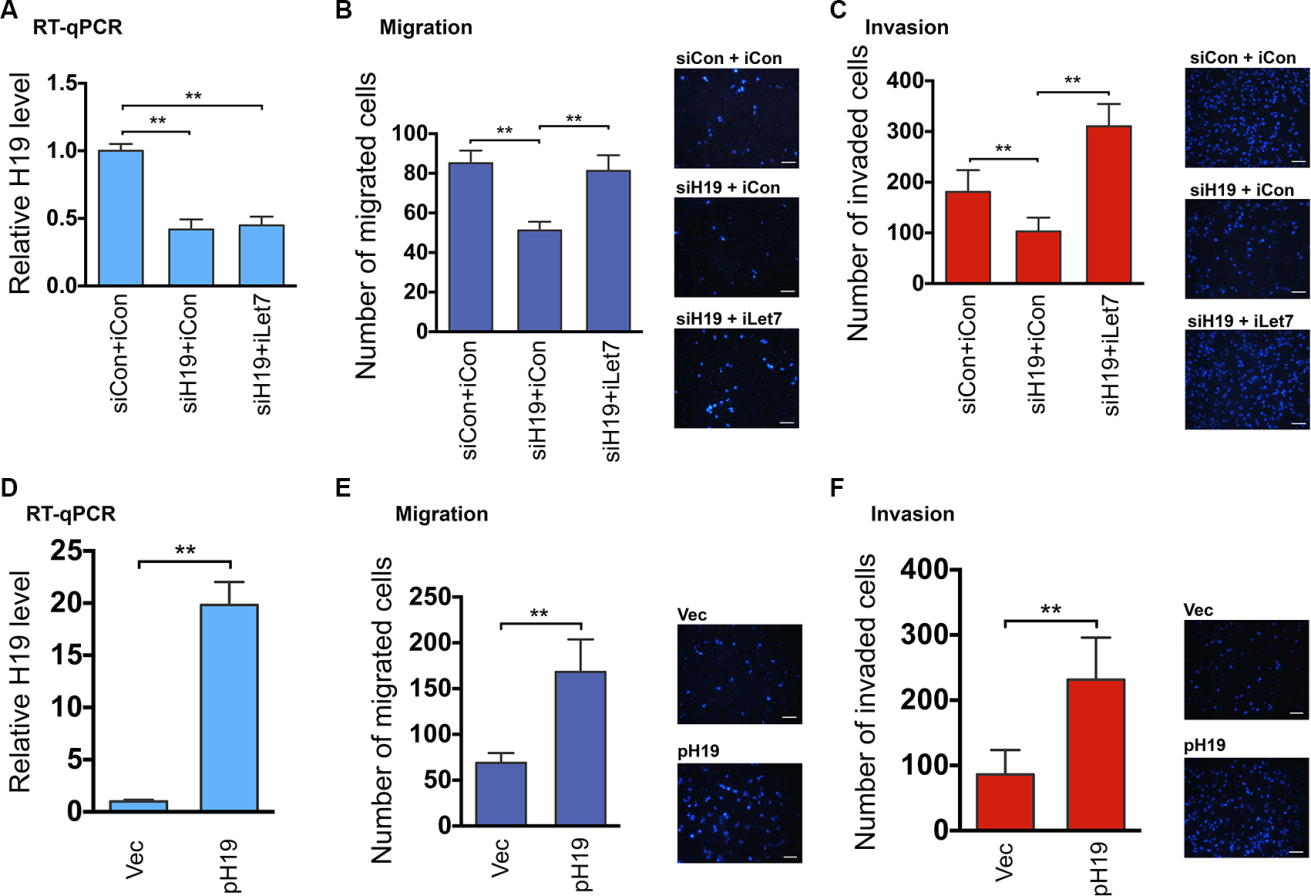
The H19/let-7 axis regulates trophoblast cell migration and invasion (**A**–**C**) HTR cells were transfected with siCon (control siRNA) plus iCon (microRNA inhibitor control), siH19 (H19-specific siRNA) plus iCon, or siH19 plus iLet-7 (let-7-specific inhibitor). RNAs were extracted 48 h post-transfection and analyzed by RT-qPCR. Relative H19 levels after normalization against beta-tubulin mRNA are presented in A. For cell migration and invasion analyses, 48 h following transfection, equal numbers of cells from each group were seeded into upper chambers. Migration and invasion were allowed to occur for 20 h and 36 h, respectively. Group t tests were performed to compare each data point with the control (siCon+iCon). Numbers are mean ± SD (*n* = 3). **p < 0.01. Representative Dapi-stained images of migrated (B) and invaded (C) cells are presented on the right. Original magnification: ×100. Size bar: 50 μm. (**D**–**F**) HTR cells were transfected with empty vector or pH19 (H19-expressing plasmid). RNAs were extracted 48 h post-transfection and analyzed by RT-qPCR (D). Results of cell migration (E) and invasion (F) are presented. Group t tests were performed to compare each data point with the control (Vec). Numbers are mean ± SD (*n* = 3). **p < 0.01.

When H19 was downregulated by siH19 (Figure 1A, compare middle column to left column), there was a concomitant decrease in cell migration (Figure 1B, compare middle column to left column) and invasion (Figure 1C, compare middle column to left column). The decreases in cell migration and invasion were not due to decreased cell proliferation and/or increased cell death (Supplementary Figure 1A). A combination of iLet7 and H19 knockdown (Figure 1A, compare right column to left column) restored both migration (Figure 1B, right column) and invasion (Figure 1C, right column) to control levels. Next, we performed reciprocal experiments by overexpressing H19. We transfected HTR with an H19-expressing plasmid pH19 [14, 16] or an empty vector as a negative control. H19 overexpression (Figure 1D) led to an increase both in migration (Figure 1E) and invasion (Figure 1F) which was not due to either increased cell proliferation and/or decreased cell death (Supplementary Figure 1B). Collectively, these results suggested that H19 promotes migration and invasion of EVT cells and that this regulation is achieved at least in part by reducing the bioavailability of let-7.

### TβR3 is a target of the H19/let-7 axis

To identify downstream genes that mediate the H19/let-7-dependent regulation of EVT, we took a genome-wide approach. HTR cells were transfected with siCon and siH19, and RNAs were extracted 48 h later and subjected to high throughput RNA deep sequencing (RNA-seq). Among the numerous genes that showed significantly altered expression as a result of H19 repression (Supplementary Data 1, and Gene Expression Omnibus (GEO) accession number GSE80237) was the type III TGF-β receptor (TβR3, also called betaglycan). The family of TGF-β cytokines (including TGF-β1, TGF-β2, and TGF-β3) signal by activating a receptor heterotetramer composed of two TβR1:TβR2 heterodimers [24] which subsequently phosphorylate (through TβR1's cytoplasmic kinase domain) the SMAD proteins which then move into the nucleus to orchestrate transcriptional responses [25]. TβR3 is unique in that its cytoplasmic domain lacks a kinase activity and that it functions as a direct regulator of TGF-β access to the signaling receptors. TβR3 binds and presents TGF-β to the TβR1:TβR2 complexes, thereby augmenting cell responsiveness to TGF-β [26]. We decided to focus on TβR3 for several reasons. First, TGF-β signaling is known to regulate EVT migration and invasion (reviewed in [8, 27]), but a role for TβR3 in these processes has not been documented. Second, human placental trophoblast cells in primary culture have been found to possess a predominant level of TβR3 and relatively lower levels of TβR1 and TβR2 [28]. Third, TβR3 has been shown to regulate invasion of non-placental cells. For example, TβR3 enhances invasion of epicardial cells through activating the Par6/Smurf1/RhoA pathway independent of SMADs [29], whereas loss of TβR3 results in decreased mesenchymal cell invasion [30]. Finally, downregulation of TβR3 expression was seen in H19 knockdown EVT cells and also in human FGR placentae where both H19 and TβR3 significantly decreased as compared to control placentae (see below).

**Figure 2:**
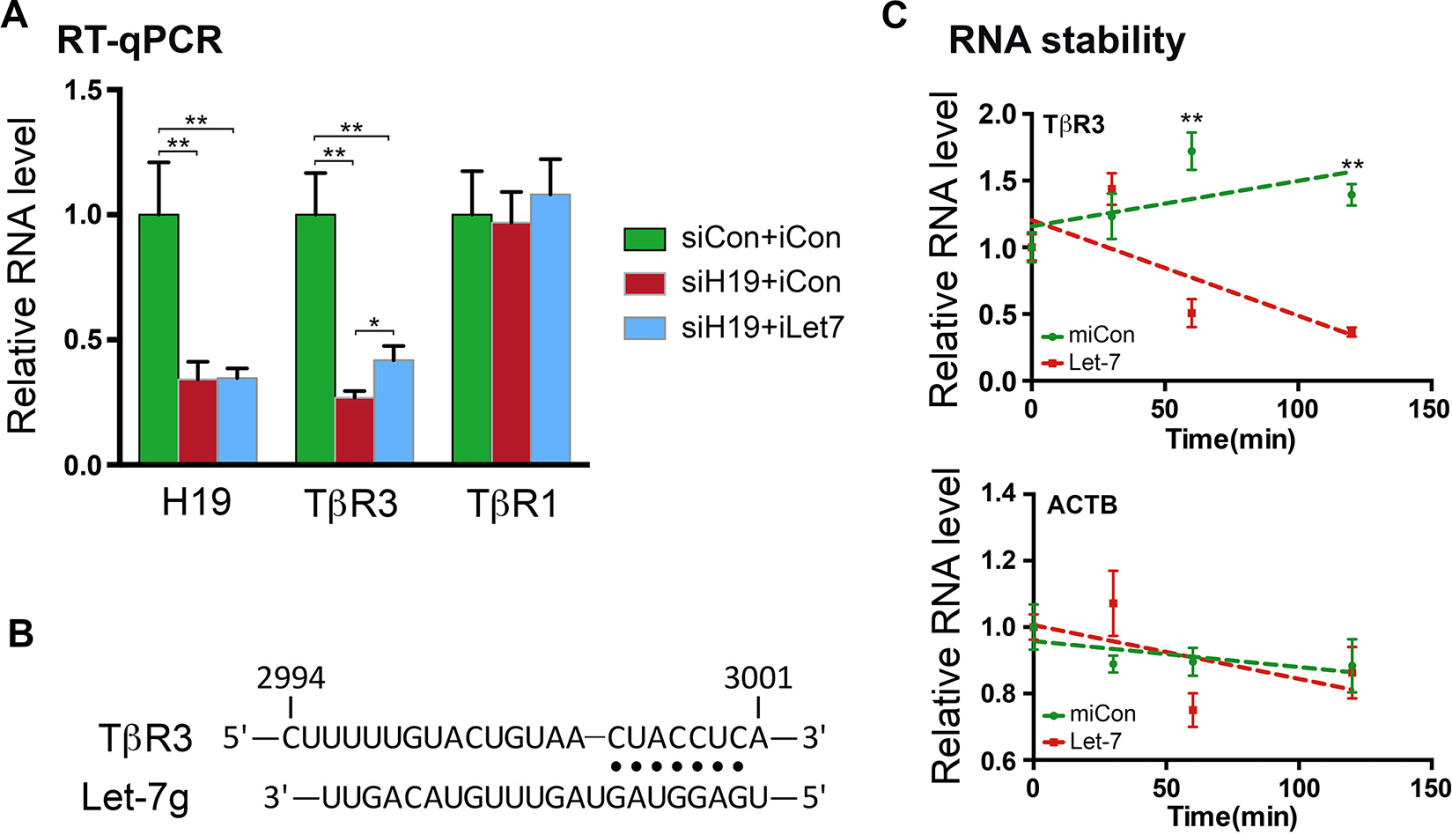
TβR3 is a target of the H19/let-7-mediated regulation (**A**) HTR cells were transfected with siCon plus iCon, siH19 plus iCon or siH19 plus iLet7. RNAs were extracted 48 h later and analyzed by RT-qPCR. The indicated RNA levels relative to those of the control (siCon+iCon, which were arbitrarily set as 1) are presented. One-sample *t* tests were performed to compare each data point with the siCon+iCon control. Numbers are mean ± SD (*n* = 3). **p < 0.01. (**B**) Schematic presentation of let-7g-binding site in TβR3 mRNA 3′-UTR. Partial sequence of human TβR3 (top) and the sequence of let-7g (bottom) are shown. Base-paired interactions between the microRNA seed region (position 2 to 8) and the target mRNA are indicated by dots. Numbers are in nucleotides relative to the transcriptional start site of TβR3. (**C**) HTR cells were transfected with Let-7 or miCon in the presence of the RNA polymerase inhibitor actinomycin D. RNAs were extracted at 0, 30, 60, and 120 minutes following incubation with the transfection mixture. TβR3 (upper panel) and beta-actin mRNA (bottom panel) levels were determined by RT-qPCR. Results are presented after normalization against beta-tubulin mRNA. Numbers are mean ± SD (*n* = 3). **p < 0.01.

When H19 was downregulated (Figure 2A, left column, compare red bar with green bar), the level of TβR3 mRNA also decreased (middle column), while that of TβR1 mRNA was not affected (right column). Importantly, iLet7 was able to partially rescue TβR3 mRNA level to that of the control (middle column, compare blue bar to red bar), consistent with TβR3 being a target of let-7 inhibition. The incomplete restoration of TβR3 mRNA level by iLet7 suggested additional regulatory mechanisms underlying TβR3 expression (see Discussion). Indeed, bioinformatic analysis predicted binding sites for multiple let-7 family microRNAs including let-7g in the 3′-UTR of TβR3 (Figure 2B and Supplementary Figure 2). Transfection of let-7 into HTR cells destabilized TβR3 mRNA (Figure 2C, top panel) but not the negative control beta-actin mRNA (bottom panel), consistent with microRNA-induced mRNA target degradation [31]. Let-7-mediated inhibition of TβR3 expression was further confirmed by Western blot analysis of cells transfected with let-7 (Supplementary Figure 3). Taken together, these results suggested that TβR3 is a target of post-transcriptional regulation by the H19/let-7 axis in EVT cells.

### TβR3 affects EVT migration and invasion through a non-canonical TGF-β signaling

**Figure 3:**
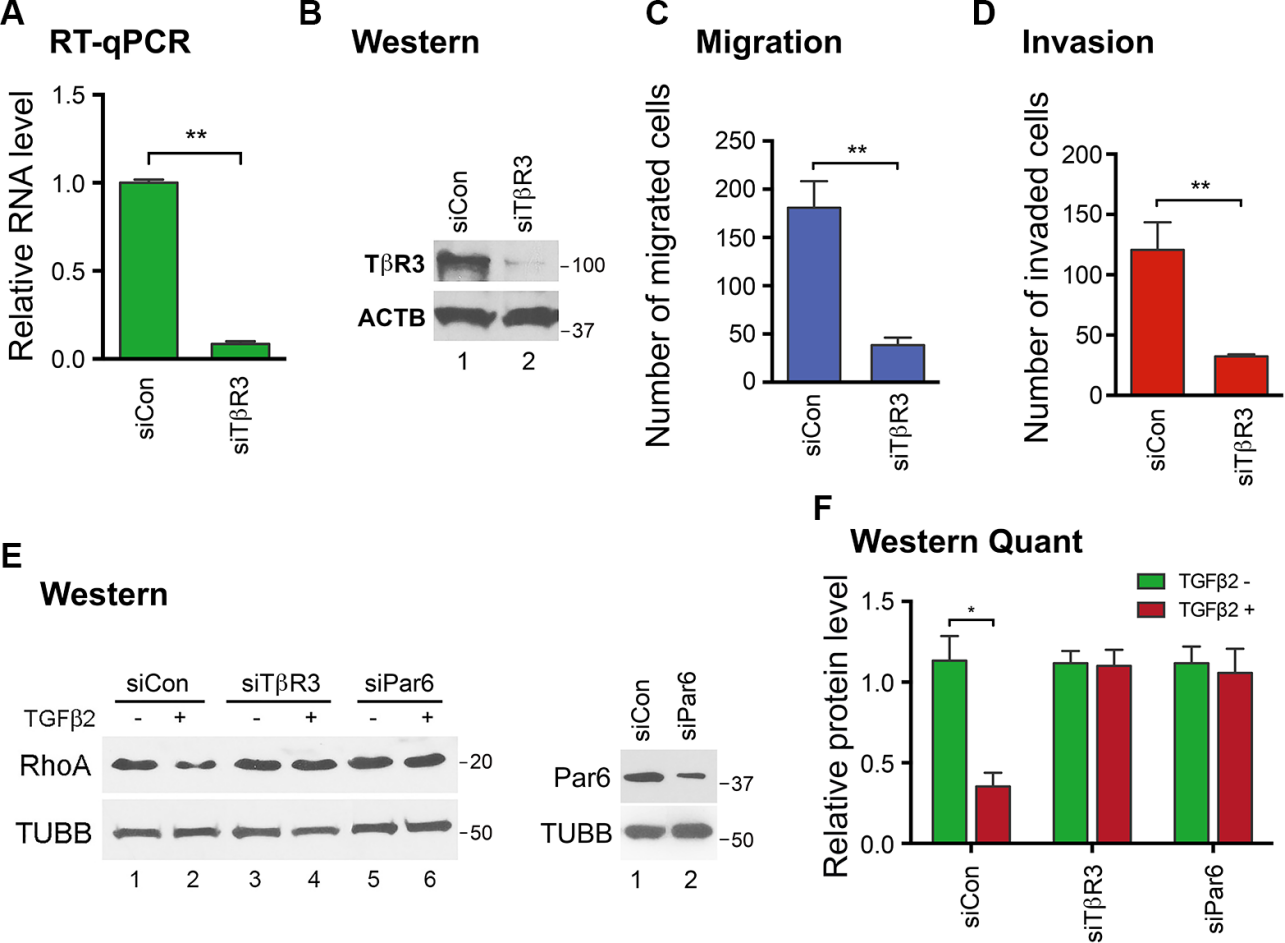
TβR3 positively impacts EVT migration and invasion through a non-canonical signaling pathway HTR cells were transfected with siCon or siTβR3. TβR3 RNA and protein levels were determined by RT-qPCR (**A**) and Western blot (**B**) analysis 48 h post-transfection. Protein molecular size markers in kDa are indicated on the right of the blots. Cell migration (**C**) and invasion (**D**) were analyzed 48 h post-transfection. Numbers are mean ± SD (*n* = 3). **p < 0.01. (**E**) HTR cells were transfected with siCon, siTβR3 or siPar6. In the left panel, at 48 h post-transfection, cells were treated with (+) or without (−)TGF-β2 for 6 h, followed by Western blot analysis to determine protein levels of RhoA (top blot) and beta-tubulin (bottom blot), which was used as a loading control. In the right panel, at 48 h post-transfection, proteins were extracted and Western blot analysis was carried out to determine Par6 protein levels. TUBB serves as a loading control. (**F**) Western blot quantifications using ImageJ from three independent transfection experiments are shown. One-sample *t* tests were performed to compare each data point with the siCon control. Numbers are mean ± SD (*n* = 3). *p < 0.05.

To confirm that TβR3 is a key downstream mediator of the H19/let-7 axis, TβR3 knockdown experiments were carried out in HTR cells and effects were evaluated. When TβR3 was reduced at both the mRNA (Figure 3A) and protein (Figure 3B) levels using a previously validated siRNA [32], cell migration (Figure 3C) and invasion (Figure 3D) also decreased without affecting cell viabilities (Supplementary Figure 4). These findings suggest that TβR3 positively impacts EVT migration and invasion. To determine the signal transduction pathway underlying the TβR3-dependent regulation, we first looked into the canonical SMAD pathway [25]. We observed no changes in SMAD phosphorylation upon TβR3 repression in HRT cells (Supplementary Figure 5). We next investigated the possible involvement of the non-canonical Par6/Smurf1/RhoA pathway previously shown to mediate TGF-β2 induced, TβR3-dependent epicardial cell invasion [29]. Upon TGF-β stimulation, TβR2 phosphorylates Par6, a key regulator of cell polarity and tight junction assembly. Activated Par6 recruits the E3 ubiquitin ligase Smurf1, which then targets RhoA for degradation [33, 34]. The Rho GTPase RhoA regulates cell motility through activation of a variety of downstream effector proteins [35]. Importantly, activation of the Par6/Smurf1/RhoA pathway which leads to RhoA degradation has been shown to be required for maintaining tumor cell motility and epicardial cell invasion [29, 33]. As TβR3 knockdown reduces EVT migration and invasion (Figure 3A–3D), we hypothesized that RhoA degradation induced by a Par6/Smurf1/RhoA signaling might be attenuated upon TβR3 knockdown. Thus, HTR cells were transfected with siCon or siTβR3, followed by TGF-β2 stimulation and RhoA protein level assessment using Western blot analysis. A decrease in RhoA was readily detectable upon TGF-β2 stimulation in siCon-transfected cells (Figure 3E, left panel, top blot, compare lane 2 to lane 1; 3F, left column, compare red bar to green bar). However, when TβR3 protein level was reduced, a RhoA decrease was no longer observed (Figure 3E, right panel, top blot, compare lane 4 to lane 3; 3F, middle column). To further support a role for this pathway, RhoA decrease was not observed (Figure 3E, right panel, top blot, compare lane 6 to lane 5; 3F, right column) in cells where Par6 protein level was downregulated (Figure 3E, right panel, compare lane 2 to lane 1) using a siRNA specific against human Par6 [36]. Taken together, these results suggested that the Par6/Smurf1/RhoA signaling is likely involved in the TβR3-mediated regulation of migration and invasion of EVT cells.

### The expression of H19 and TβR3 is decreased in FGR placentae

**Figure 4:**
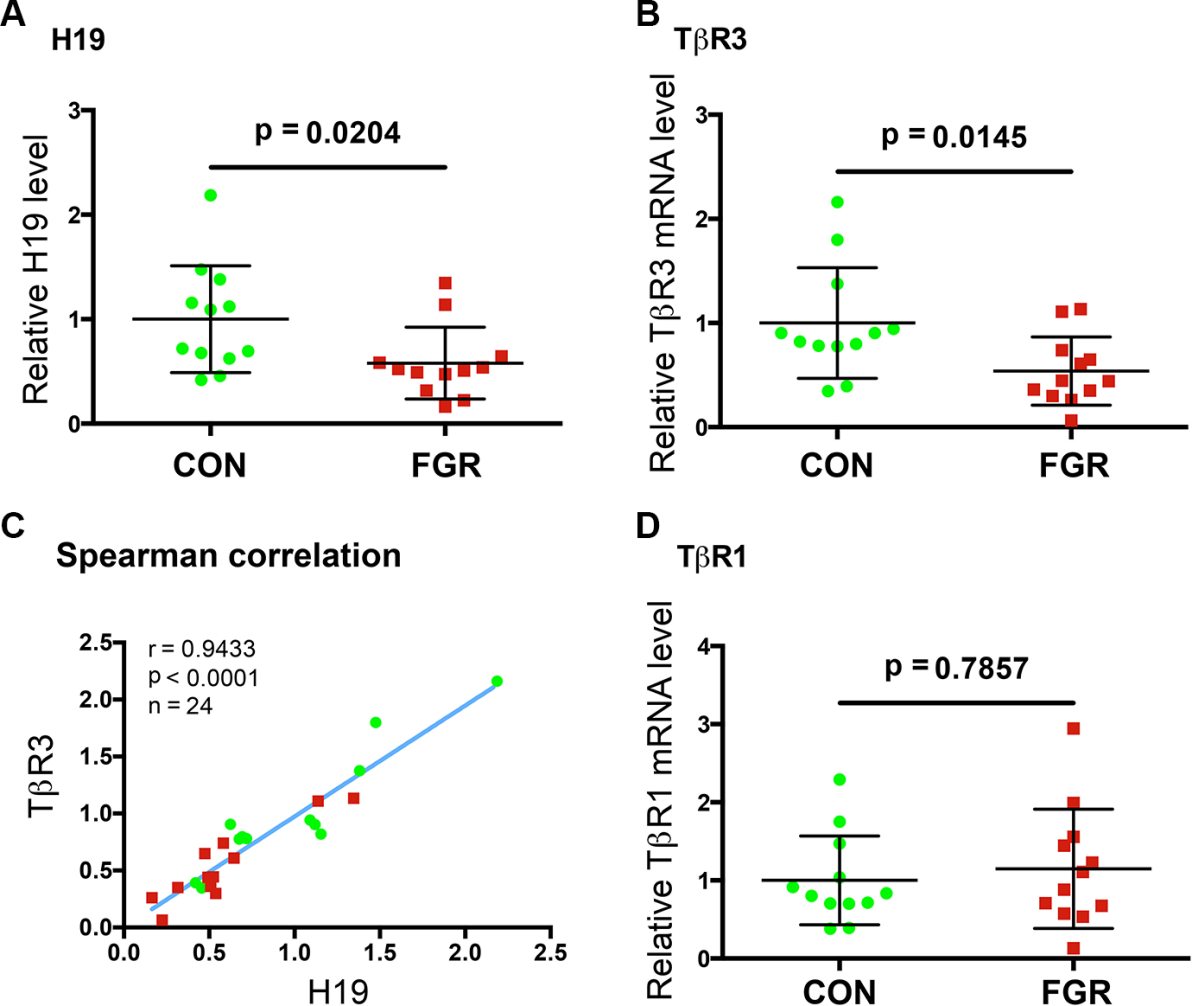
The expressions of H19 and TβR3 are significantly decreased in the FGR placentae (**A, B, D**) Scatter plot of RNA levels determined by RT-qPCR. The horizontal line represents group median, and the whiskers mark the interquartile range (**C**) Spearman correlation suggests an *in vivo* positive correlation between the expressions of H19 and TβR3 in a statistically significant manner. Spearman correlation coefficient, *p* values, and sample numbers are marked on the left top of the plot.

Given that failure of trophoblast invasion is associated with FGR [7, 8] and that H19 positively regulates trophoblast cell migration and invasion through TβR3-mediated signaling (current work), we sought to determine whether the expression of H19 and TβR3 might be altered in human FGR placentae. Thus, placental tissue was collected from pregnancies with idiopathic preterm FGR (*n* = 12) and gestational age-matched controls with spontaneous preterm delivery (*n* = 12) (Patient characteristics in Supplementary Table S1). RNAs were isolated and gene expression levels were determined by RT-qPCR. Mann-Whitney *U* test revealed a significant H19 decrease in the FGR placentae (FGR) compared to the control (CON) group (Figure 4A, *p* = 0.024,). Not surprisingly, there was a parallel decrease in TβR3 in the FGR compared to the CON group (Figure 4B, *p* = 0.0145,). Spearman correlation showed a positive relationship between H19 and TβR3 (Figure 4C, *p* < 0.0001), suggesting an *in vivo* functional interaction between H19 and TβR3. Notably, there was no significant difference in the expression of TβR1 between the two groups (Figure 4D). Together, these results suggested that decreased expression of H19 and TβR3 might contribute to impaired migration and invasion of trophoblast cells during placental development in pregnancies complicated by FGR.

## DISCUSSION

**Figure 5:**
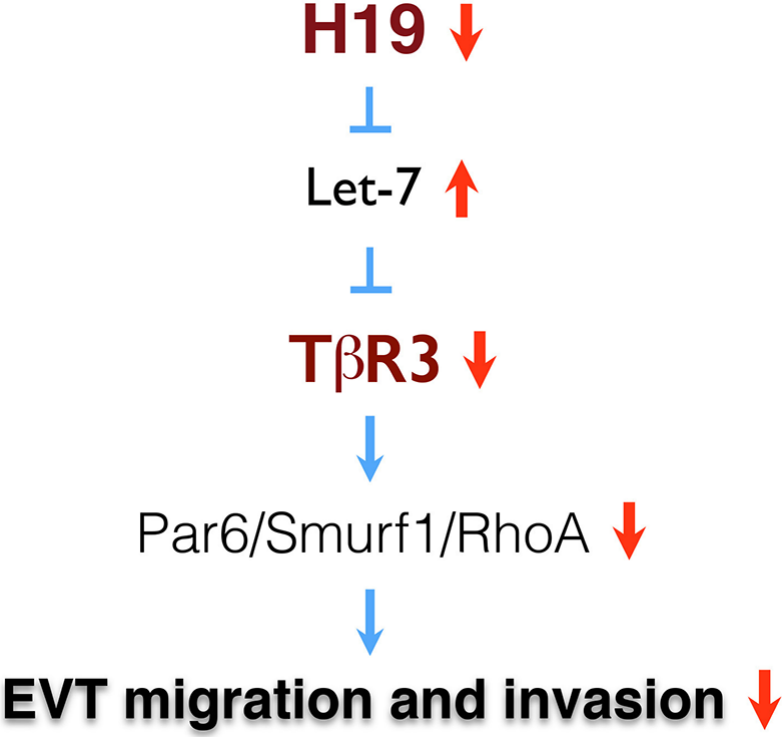
A model of H19/TβR3-mediated signaling in the regulation of EVT migration and invasion

In the present study we show that H19 repression decreases TGF-β signaling via the Par6/Smurf1/RhoA pathway activated by TβR3, leading to impaired migration and invasion of EVT cells (Figure 5). We provide evidence that dysregulation of H19/TβR3 signaling may contribute to the underlying mechanism of idiopathic FGR.

The role of different, individual TGF-β cytokine isoforms in regulating EVT migration and invasion has been studied for many years (reviewed in [8, 27]). Recently, imbalanced TGF-β signaling through two TβR1 receptor subtypes (ALK5 and ALK1) has been suggested as a pathogenic cause of FGR [37]. In this work, the authors presented evidence for altered TGF-β signaling via the canonical SMAD pathway, leading to an elevated sphingosine:ceramide ratio found in FGR placentae. They postulated that this finding explains the augmented trophoblast cell death rates and impaired placentation seen in FGR. However, they also noted that the canonical SMAD pathway does not entirely explain their findings and suggest a role for a non-redundant mechanism by which TGF-β signaling is altered in cases of FGR. Our study uncovers a novel pathway, Par6/Smurf1/RhoA, which is activated by TβR3 and regulates trophoblast cell migration and invasion. It is possible that both the canonical and the non-canonical pathways play critical and non-redundant roles in placentation. It remains to be investigated whether the two pathways cross-talk and whether they may act in concert to regulate trophoblast cell function.

While believing that TβR3 is one downstream mediator of the H19/let-7-mediated regulation, our RNA-seq data revealed numerous gene expression changes as a result of H19 knockdown (Supplementary Data 1). The partial rescue of TβR3 expression by iLet7 (Figure 2A, middle column) versus the full restoration of migration and invasion to the siH19 cells by iLet7 (Figure 1A–1C) implicates other let-7-targeted genes in the H19-mediated regulation. The incomplete restoration of TβR3 expression by iLet7 was not surprising given that H19 is a multifunctional lncRNA. We have recently reported that H19 alters gene methylation genome-wide through modulation of the S-adenosylhomocysteine hydrolase activity [23], including H19 knockdown-induced hypomethylation of TβR3 in the intron region [23]. It remains to be tested whether downregulation of H19 in human trophoblast cells might induce methylation changes in the TβR3 gene, which may explain the partial restoration of TβR3 expression by iLet7. Our findings represent the first example of a lncRNA-based mechanism of FGR and hold promise for the development of novel predictive, diagnostic and therapeutic modalities for idiopathic FGR.

## MATERIALS AND METHODS

### Materials

HTR-8/SVneo cells were a generous gift from Charles Graham (Queen's University, Kingston, Canada) and were cultured in RPMI1640 (Gibco, 11965-092) supplemented with 10% fetal bovine serum, heat inactivated, 1% penicillin/streptomycin, and 1% L-glutamine. Antibodies for TβR3 (Abcam, ab166705; used at a dilution of 1/500), Par6 (Abcam, ab180159; used at a dilution of 1/1000), TUBB (Abcam, ab6046; used at a dilution of 1/10000), ACTB (Cell Signaling, 4967; used at a dilution of 1/10000), RhoA (Cell Signaling, 2117; used at a dilution of 1/1000), SMAD2/3 (Cell Signaling, 3102; used at a dilution of 1/1000), and phosphorylated SMAD2/3 (Cell Signaling, 3101; used at a dilution of 1/1000) were purchased. siH19 (Ambion, 4390816/n272452), siTβR3 (Dharmacon, L-010545-00), siPar6 (Ambion, 4392420/s27161), siCon (Ambion, Am4636), iLet7 (Ambion, 4392431), iCon (Ambion, AM17010), let-7 mimics (let-7) (Ambion, AM17100/PM10050) and miCon (Ambion, AM17110) were purchased. Plasmids expressing human H19 (pH19) and empty vector were previously described [14, 16]. TGF-β2 cytokine was purchased (Cell Signaling, 8406).

### siRNA knockdown, iLet-7 rescue, and H19 overexpression experiments

Cells were transfected in a 48-well plate scale. To prepare siRNA transfection solution for each well, 15 pmol of siCon (or siH19, siPar6, siTβR3) was mixed with 50 μl OPTI-MEM by gentle pipetting. In parallel, 0.5 μl Lipofectamine 2000 was mixed with 50 μl OPTI-MEM. Following 5 minutes of incubation at room temperature (RT), the two were mixed by gentle pipetting and incubated for 20 to 30 minutes at RT to allow siRNA/lipid complexes to form. At the end of incubation, the 200 μl transfection solution was used to re-suspend the cell pellet (~4 × 10^4^ cells/well). After incubation at RT for 10 minutes, regular growth medium was added at a ratio of 1:3 (1 volume of transfection solution/2 volumes of growth medium) and the cell suspension was transferred to the culture plate. After 24 h incubation at 37°C in 5% CO_2_, the medium was replaced with fresh growth medium. RNAs and proteins were extracted and analyzed at the indicated time points following transfection.

Plasmid DNA transfections were carried out as described for siRNA, except that 0.8 μg DNA in 50 μl OPTI-MEM and 0.5 μl Lipofectamine 2000 in 50 μl OPTI-MEM were used for each well of cells (the 100 μl of final transfection reagent with 400 μl of regular growth medium were added to each well). For iLet7 rescue experiments, 15 pmol of siCon/siH19 and 45 pmol of iCon/iLet7 were used for each well of cells.

### RNA extraction and RT-qPCR

Total RNAs were extracted from cells using PureLink RNA Mini Kit (Ambion, catalog number 12183018A). cDNA was synthesized using Bio-Rad iSCRIPT kit (1725122) in a 20 μl reaction containing 100–500 ng of total RNA. Real-time quantitative PCR was performed in a 15 μl reaction containing 0.5–1 μl of cDNA using iQSYBRGreen (Bio-Rad) in a Bio-Rad iCycler. PCR was performed by initial denaturation at 95°C for 5 min, followed by 40 cycles of 30 sec at 95°C, 30 sec at 60°C, and 30 sec at 72°C. Specificity was verified by melting curve analysis and agarose gel electrophoresis. The threshold cycle (Ct) values of each sample were used in the post-PCR data analysis. The real-time PCR primers are listed in Supplementary Table 2.

### Western blot analysis

Cell pellets were quickly lysed in 5 volumes of 2× SDS-sample buffer heated at 100°C for 5 min, with occasional vortexing. Five to 10 μl of homogenized samples were loaded onto 10% SDS gel, followed by Western blot analysis. The linear dynamic range of each protein of interest was determined by serial dilutions. Bands on Western blot gels were quantified using ImageJ.

### Let-7 transfection and TβR3 RNA and protein analysis

To provide further evidence that TβR3 is a target of let-7, let-7 mimics were transfected into HTR cells in a 48-well plate scale, followed by RNA and protein analyses 6 h and 48 h later, respectively. To analyze let-7 effects on RNA stability, let-7 transfection combined with actinomycin D time course analysis was performed. To prepare transfection cocktail, 1 pmol of control miRNA (miCon) or let-7a mimic was mixed with 50 μl of OPTI-MEM. In parallel, 0.5 μl of Lipofectamine 2000 was mixed with 50 μl of OPTI-MEM. Following 5 min of incubation, the two solutions were mixed and incubated at RT for 20 min. The resulting 100 μl of transfection cocktail was added to HTR cells pre-washed with OPTI-MEM. Upon adding the transfection cocktail, actinomycin D was also added to each well at a final concentration of 10 μg/ml. Total RNA was extracted at the indicated time points, followed by RT-qPCR analysis. Results are presented after normalization against beta-tubulin mRNA levels with 0 time point RNA levels arbitrarily set as 1. For TβR3 western blot analysis, cells harvested at 48 h post-transfection were lysed in 5 volumes of 2× SDS-sample buffer heated at 100°C for 5 min. Five to 10 ml of homogenized samples were loaded onto 10% SDS gel, followed by Western blot analysis as described above.

### Quantitative cell migration and invasion assays

These were carried out as previously described [38] with minor modifications. Briefly, transwell chambers (Corning, 3422, 8-μm pores) placed into a 24-well plate (Fisher Scientific, 07200150,) were used in the assays. The lower chamber was filled with 500 μl RPMI 1640 containing 20% FBS. Cells were trypsinized, counted, and re suspended in serum-free RPMI. For migration assays, 5 × 10^4^ cells in 200 μl serum-free RPMI were added to the upper chamber. Equal numbers of cells from experimental and control groups were loaded into the upper chamber. The cells were allowed to migrate for 20 h at 37°C before fixing. For invasion assays, 100 μl Matrigel (BD Biosciences, Bedford, MA, USA), 1:3 diluted in serum-free RPMI, was coated onto the upper chamber and incubated at 37°C for 2 h. Equal numbers of cells from experimental and control groups were seeded into the upper chamber at a concentration of 8 × 10^4^/200 μl and incubated for 36 h at 37°C before fixing. The non-migrated cells were removed from the upper surface of the membrane by scraping with a cotton swab. Cells on the bottom surface of the membrane were fixed with 95% ethanol at RT for 30 min, gently rinsed with phosphate-buffered saline (PBS), stained with Dapi for 5 min, and photographed using a Zeiss (Melville, NY, USA) microscope system. Migration/invasion was assessed by counting the number of stained cell nuclei from 5 random fields per filter in each group at ×100 magnification. The experiments were conducted in triplicate. Cell counts were expressed as the mean number of cells per field of view.

### Cell viability analysis

To prepare for cell viability analysis, cells were seeded at 48 h post-transfection in 96-well plates at a density of 1 × 10^4^/well at the same time when migration and invasion assays were initiated. Cells were allowed to grow for 36 h and viabilities were determined using the Cell Titer Blue Cell Viability kit (Promega) according to the manufacturer's protocols.

### Cytokine treatment

Cells were transfected with siCon, siTβR3 or siPar6 in 48-well plates as described above. The medium was changed into serum-free RPMI 1640 at 24 h post-transfection. TGF-β2 was applied to the cell 48 h post-transfection at a final concentration of 20 ng/ml in serum-free RPMI 1640. Six hours later, protein was extracted, followed by Western blot analysis.

### Study population

We studied placenta samples from 24 women enrolled in the following groups:

idiopathic fetal growth restriction (FGR, *n* = 12) and preterm control (CON, *n* = 12). Placentae were collected under protocols approved by the Human Investigation Committee of Yale University. Informed consent was obtained from all participants prior to enrollment. Gestational age was established based on menstrual dating confirmed by sonographic examination prior to 20 weeks' of gestation. Characteristics of the study population are shown in Supplementary Table 2.

The CON group consisted of placentae from pregnancies with spontaneous preterm labor and/or preterm premature rupture of membranes without evidence of clinical nor histological chorioamnionitis. FGR was defined as weight < 10th percentile for gestational age at birth in pregnancies not complicated by preeclampsia. A detailed description of these patients was previously reported [39–41].

### Tissue collection and analysis of gene expression

Following delivery, placentae were brought immediately to the laboratory. Approximately 1 g of placental villous tissue from the central region of the placenta (dissected free from decidua basalis) was collected, frozen in liquid nitrogen, and maintained at −80°C. RNA was extracted from placental tissue using Trizol reagent (Life Technologies, Grand Island, NY). cDNA was synthesized from 3–5 μg RNA using oligo-deoxythymidine 12–18 (catalog item 18418-012; Invitrogen) and SuperScript II reverse transcriptase (catalog item 18064-014; Invitrogen) in a 20 μl reaction volume according to the manufacturer's instructions. qPCR was performed as described above using beta-tubulin mRNA as a loading control.

### Statistical analysis

Patient characteristics and *in vivo* gene expression data are presented as median and interquartile range (IQR) and were analyzed using Mann-Whitney *U*-test. Spearman correlations were performed for gene co-expression analyses. *In vitro* data are presented as mean ± standard deviation (SD) and analyzed using two-tailed Student *t* test. Statistical analyses were performed using the Statistical Package for the Social Science (SPSS) computer software version 17.0 (IBM SPSS Statistics, Chicago, IL, USA). Figures were constructed using Prism 6 version 6.0f (GraphPad Software, Inc.). *P*-values < 0.05 were considered significant.

## SUPPLEMENTARY MATERIALS FIGURES AND TABLES




