# Supplementary material for: H19 long noncoding RNA alters trophoblast cell migration and invasion by regulating TβR3 in placentae with fetal growth restriction
Source: Oncotarget. 2016 May 21;7(25):38398–407. doi: 10.18632/oncotarget.9534 (PMC5122399; doi:10.18632/oncotarget.9534)
Supplement: Supplementary file 1 [file oncotarget-07-38398-s001.pdf]

H19 long noncoding RNA alters trophoblast cell migration and invasion by regulating TβR3 in placentae with fetal growth restriction

Supplementary Materials

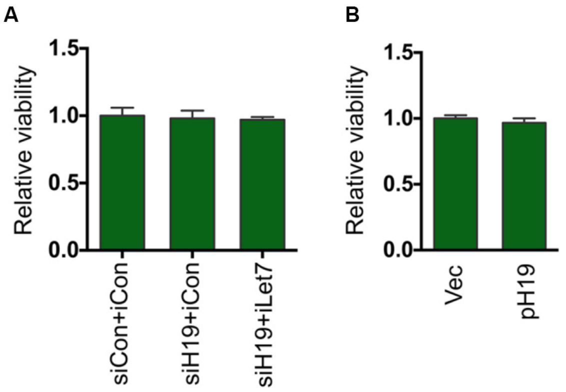

Supplementary Figure S1: Cell viability. HTR cells were transfected with (**A**) siCon plus iCon, siH19 plus iCon or siH19 plus iLet7, or (**B**) Vec or pH19. Forty-eight hours later, cells were detached from the plate and seeded into 96-well plates at a density of 1×10^4/well at the same time when migration and invasion assays were initiated. Cells were allowed to grow for 36 h and viabilities were determined. The relative viabilities are presented with those of the control groups (siCon+iCon or Vec) arbitrarily set as 1.

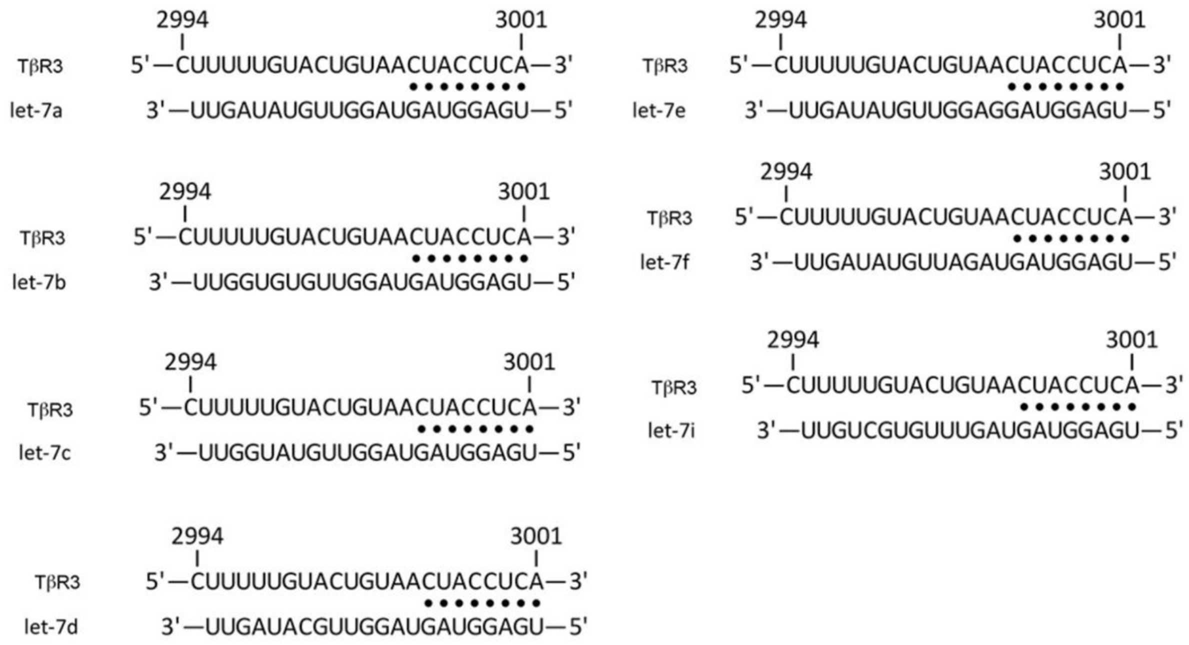

Supplementary Figure S2: Schematic presentation of let-7-binding sites in TβR3 mRNA 3′-UTR. Partial sequence of human TβR3 (top) and the sequences of let-7 suptypes (bottom) are shown. Base-paired interactions between the microRNA seed region (position 2 to 8) and the target mRNA are indicated by dots. Numbers are in nucleotides relative to the transcriptional start site of TβR3.

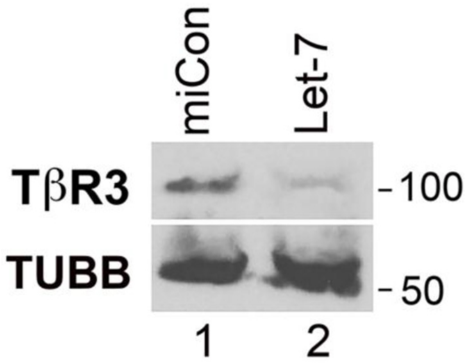

Supplementary Figure S3: HTR cells were transfected with miCon or Let-7, followed by western blot analysis at 48 h post-transfection. TUBB was used as a loading control. Protein markers in kDa are show on the right.

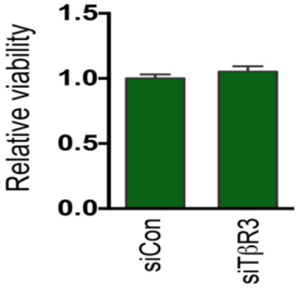

Supplementary Figure S4: Cell viability. HTR cells were transfected with siCon or siTβR3. Forty-eight hours later, cells were detached from the plate and seeded into 96-well plates at a density of 1×10^4/well at the same time when migration and invasion assays were initiated. Cells were allowed to grow for 36 h, followed by viability analysis. The relative viabilities are presented with that of the siCon group arbitrarily set as 1.

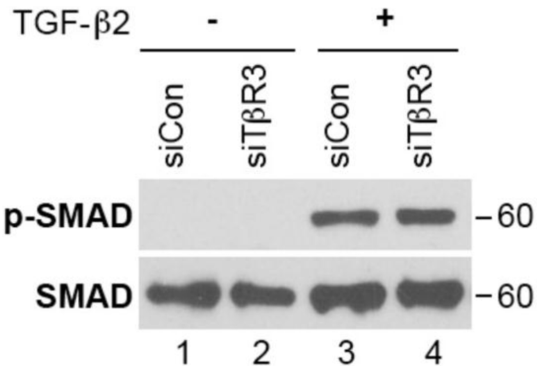

Supplementary Figure S5: TβR3 knockdown does not affect SMAD-mediated signaling. HTR cells were transfected with siCon, or siTβR3. At 48 h post-transfection, cells were treated with (+) or without (−) TGF-β2 for 6 h, followed by Western blot analysis to determine protein levels of phosphorylated SMAD2 (p-SMAD) and total SMAD2 (SMAD). Molecular size markers in kDa are indicated on the right.

Supplementary Table S1: Characteristics of the study population

Variables	CON<br>*n* = 12	IUGR<br>*n* = 12	*P* value
**Demographic and clinical characteristics**			
Age, *years*†	28 [23 – 32]	27 [20 – 34]	0.931
Gravidity†	2 [1 – 3]	2 [1 – 3]	0.582
Parity†	0 [0 – 1]	0 [0 – 1]	0.801
Non Caucasian race‡	3 (25)	6 (50)	0.400
Maternal body mass index†	32 [22 – 45]	25 [20 – 29]	0.202
Gestational age at delivery, *weeks*†	31 [30 – 33]	30 [29 – 34]	0.862
Birthweight, grams†	1,783 [1,358 – 1,953]	880 [695 – 1,445]	0.006
PPROM‡	6 (50)	0 (0)	0.014
1 minute Apgar†	7 [6 – 8]	8 [2 – 9]	1.000
5 minute Apgar†	9 [8 – 9]	9 [7 – 9]	0.674
Cesarean delivery†	5 (42)	11 (92)	0.027
**Umbilical arterial Doppler assessment**			
Resistivity index	NA	0.91 [0.78 – 0.97]	NA
Pulsatility index	NA	2.1 [1.4 – 2.5]	NA
S/D ratio	NA	4.4 [3.5 – 16.0]	NA
Absent End-Diastolic Flow	NA	12 (100)	NA

†Data presented as median [interquartile range] and analyzed by Mann-Whitney *U* test.
‡Data presented as *n* (%) and analyzed by Fisher's exact tests.
PPROM: preterm premature rupture of the membranes; S/D ratio: systolic/diastolic ratio.

Supplementary Table S2: Real-time PCR primer sequences

Gene	Forward primer	Reverse primer
H19	5′-ACTCAGGAATCGGCTCTGGAA-3′	5′-CTGCTGTTCCGATGGTGTCTT-3′
β-Tubulin	5′-CGTGTTCGGCCAGAGTGGTGC-3′	5′-GGGTGAGGGCATGACGCTGAA-3′
β-Actin	5′-ATCAAGATCATTGCTCCTCCTGAG-3′	5′-CTGCTTGCTGATCCACATCTG-3′
TβR1	5′-CCTCACTGAGAGGGAACAGA-3′	5′-AAGATGATCTCCAGCACAGC-3′
TβR3	5′-CTGGCCAGCTACAGAGAGAG-3′	5′-ACCCTCAGACACCAAAAACA-3′

Supplementary Data S1: Galaxy1121-(Cuffdiff_on_data_28)
